# Crosstalk between miRNAs and DNA Methylation in Cancer

**DOI:** 10.3390/genes14051075

**Published:** 2023-05-12

**Authors:** Michela Saviana, Patricia Le, Lavender Micalo, Daniel Del Valle-Morales, Giulia Romano, Mario Acunzo, Howard Li, Patrick Nana-Sinkam

**Affiliations:** Department of Internal Medicine, Division of Pulmonary Diseases and Critical Care Medicine, Virginia Commonwealth University, 1250 E. Marshall Street, Richmond, VA 23298, USA

**Keywords:** DNA methylation, miRNA, cancer

## Abstract

miRNAs are some of the most well-characterized regulators of gene expression. Integral to several physiological processes, their aberrant expression often drives the pathogenesis of both benign and malignant diseases. Similarly, DNA methylation represents an epigenetic modification influencing transcription and playing a critical role in silencing numerous genes. The silencing of tumor suppressor genes through DNA methylation has been reported in many types of cancer and is associated with tumor development and progression. A growing body of literature has described the crosstalk between DNA methylation and miRNAs as an additional layer in the regulation of gene expression. Methylation in miRNA promoter regions inhibits its transcription, while miRNAs can target transcripts and subsequently regulate the proteins responsible for DNA methylation. Such relationships between miRNA and DNA methylation serve an important regulatory role in several tumor types and highlight a novel avenue for potential therapeutic targets. In this review, we discuss the crosstalk between DNA methylation and miRNA expression in the pathogenesis of cancer and describe how miRNAs influence DNA methylation and, conversely, how methylation impacts the expression of miRNAs. Finally, we address how these epigenetic modifications may be leveraged as biomarkers in cancer.

## 1. Introduction

The term “epigenetics” was first coined by Conrad Hal Waddington and refers to the mechanisms of inheritance in addition to standard genetics [1]. To date, epigenetics indicates all mechanisms that can regulate gene activity without directly affecting the DNA sequence [2]. Epigenetic mechanisms include histone modifications, DNA methylation, and microRNAs (miRNAs/miRs). Histone modifications affect the chromatin structure and function, facilitating or inhibiting the accessibility of the transcription machinery [3]. DNA methylation and miRNAs, similarly, silence specific gene expression. While DNA methylation provides stable gene silencing at the transcriptional level, miRNAs inhibit gene expression at the post-transcriptional level. Both DNA methylation and miRNA expression have been found to be dysregulated in several human diseases including cancers [4,5]. Intriguingly, in the past several years, the mutual regulation between these two epigenetic mechanisms has been increasingly recognized [6], uncovering a new level of complexity in the regulation of gene expression in cancer. These two mechanisms for epigenetic regulation can impact distinct biological processes, including metastasis, apoptosis, cell proliferation, and induction of senescence. Here we review the crosstalk and mutual regulation between DNA methylation and miRNAs and how their dysregulation is involved in human cancers.

## 2. Mechanisms of DNA Methylation

DNA methylation is a modification that results in the transcriptional silencing of gene expression, transposon silencing, and X chromosome inactivation while maintaining genomic stability. DNA methylation occurs either as maintenance during DNA replication or de novo at CpG regions of the chromatin. In mammals, the three main methyltransferases involved in DNA methylation are DNMT1, DNMT3A, and DNMT3B [7]. DNMT1 maintains DNA methylation of the daughter strand during DNA replication [8,9]. DNMT1 is normally present at an auto-inhibitory state where the replication foci targeting sequence (RFTS) domain is buried into its methyltransferase domain. During DNA replication, Ubiquitin-like containing PHD and RING finger domains 1 (UHRF1) binds to hemi-methylated CpG dinucleotides at the replication fork [10] and to the histone H3 modification H3K9me2 and H3K9me3 [11]. UHRF1 ubiquitinates the H3 tail and then recruits DNMT1, releasing the RFTS domain from the active site of DNMT1 [12]. DNMT1 methylates the daughter strand of the replicating DNA [12].

DNMT3A and DNMT3B catalyze de novo DNA methylation at various CpG locations throughout the genome [13,14]. The site of de novo DNA methylation is dictated by the histone H3 methylation status. The ATRX-DNMT3A-DNMT3L (ADD) domain of DNMT3BA and DNMT3B acts as an auto-inhibitory domain of methylation activity; the ADD domain binding abolishes the inhibition to unmethylated H3K4 [15,16]. The Pro-Trp-Trp-Pro (PWWP) domain of DNMT3BA and DNMT3B binds to histones H3K36me2 and H3K36me3 [17]. CpG-rich promoters of actively transcribed genes are modified with H3K4me3, which repels ADD domain binding and prevents DNA methylation at these promoters [15].

Additionally, DNA methylation can be removed by Ten-eleven Translocation (TET) enzymes, TET1, TET2, and TET3 [18]. The TET enzymes oxidize 5mC down to 5-hydroxymethylcytosine (5hmC), 5-formylcytosine (5fC), and 5-carboxylcytosine (5caC) [19]. The oxidized forms of 5mC are not recognized by the methylation maintenance mechanism and are subsequently removed after DNA replication [20].

**Figure 1:**
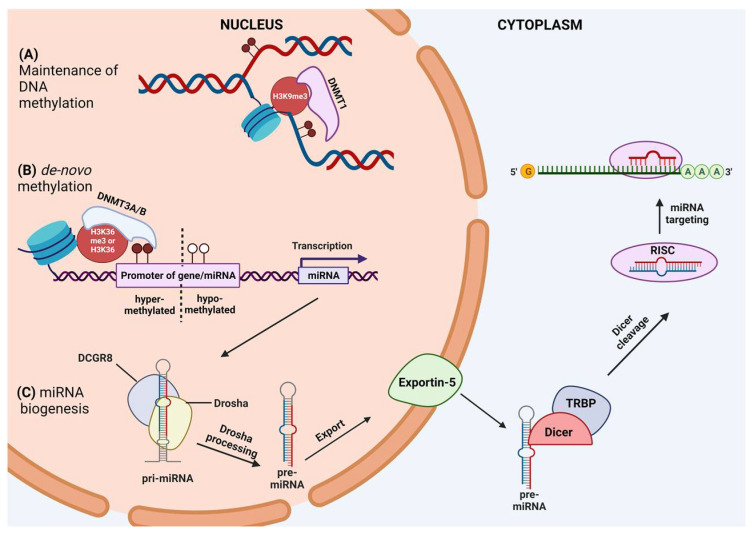
Schematic representation of the mechanism of DNA methylation and miRNA biogenesis. (**A**) Maintenance of methylation occurs during DNA replication. The histone H3 modification H3k9me3 recruits DMNT1 to methylate the daughter strand. (**B**) De novo methylation occurs at CpG locations throughout the genome. The histone H3 modification H3K36me3 inhibits the methylation activity of DNMT2/3. Unmethylated H3K36 recruits DNMT3A/B to the CpG sites, causing its hypermethylation. (**C**) miRNA biogenesis starts in the nucleus where the pri-miRNA is synthesized and then cropped by Drosha/DGCR8, converting into pre-miRNA. Exportin 5 mediates the pre-miRNA transport from the nucleus to the cytoplasm where it is processed by Dicer, producing a mature miRNA duplex of 22 nucleotides. Mature miRNA is loaded in the RISC complex that unwinds the duplex. The passenger strand is expulsed, while the guide strand is retained in the RISC complex that coordinates the interaction between the miRNA and its mRNA target. Created with BioRender, https://www.biorender.com/ (accessed on 8 May 2023).

Rearrangement of DNA methylation patterns occurs during embryonic development [21] and stem cell differentiation [22], particularly in neuronal development [23]. Specific methylation patterns vary between cell types, and germline-specific genes are silenced through DNA methylation in somatic cells [24]. Disruptions of DNA methylation patterns are a hallmark of cancer, where demethylation of oncogenes and hypermethylation of tumor suppressors promote the cancerous phenotype [4]. In particular, the methylation patterns of miRNAs are dysregulated in various cancers. A representation of the DNA methylation mechanism is schematized in Figure 1.

## 3. miRNA: Biogenesis and Function

Over the last three decades, miRNAs have been the most studied non-coding RNAs, given their integral role in fundamental biological processes [25]. They are 18–24 nucleotides (nt) in length and regulate gene expression silencing of mRNA(s) at the post-transcriptional level. miRNAs have a biogenesis pathway distinct from those of long non-coding (lncRNA) and use common cellular RNA transcription and maturation mechanisms [26,27]. The transcriptional units that generate miRNAs are isolated or clustered and localized to coding gene, non-coding gene, and intergenic regions [26]. RNA polymerase II transcribes all canonical miRNAs in Metazoa, creating a primary transcript called “pri-miRNA” that is >1000 nts long. Structurally, this transcript has at least one loop to form a hairpin essential for the Microprocessor complex. This is a multiprotein complex formed by the endonuclease Drosha and two molecules of DiGeorge syndrome critical region 8 protein (DGCR8), a double-stranded RNA binding protein [28,29,30] that cuts the pri-miRNA into a pre-miRNA (~70 nts). Note that all of the canonical pri-miRNAs have a 5′ cap but not a Poly(A) tail because processing by Microprocessor can be activated in a “non-canonical” manner [31]. At this point, the pre-miRNA, after binding the Exportin5 (EXP5) and RAN-GTP complex, migrates into the cytosol through a nuclear pore complex. After GTP hydrolysis, the EXP5/RAN complex is disassembled, and the pre-miRNA is released [32]. The pre-miRNA is further processed in the cytosol by DICER, another RNAse III endonuclease in the miRNA duplex, which contains the mature miRNA paired to its passenger strand [33]. Dicer’s partner protein, called TRBP in mammals, is important but not essential for miRNA maturation. This duplex is loaded with Argonaute proteins (AGOs) and chaperone proteins to form the silencing complex called RNA-Induced Silencing Complex (RISC). The duplex unwinds, and the passenger strand is expulsed; at this point, RISC is ready to carry out its function [34]. The loaded miRNA strand functions as a “guide” by base pairing with its target mRNAs, thereby inducing translational repression, mRNA deadenylation, and mRNA decay [35]. Usually, the miRNA-binding site is located in the 3′ untranslated region (3′UTR) of the mRNA, but there is evidence that miRNAs can also bind to the coding sequence (CDS) of target mRNA and 5′UTR [36,37]. The “seed region” of miRNAs spans between 2 and 7 nts and is crucial for recognizing target mRNAs [38]. It has been demonstrated that a single mismatch in the seed region can compromise the binding and targeting of the miRNA to mRNA [39]. The mRNA target sequences are termed miRNA response elements (MREs). There is usually a lack of a fully complementary miRNA:MRE interaction in animals, given the presence of at least central mismatches [40,41]. The miRISC complex summons GW182 family proteins that are essential as a scaffold for other effector proteins, including the poly(A)-deadenylase complexes PAN2-PAN3, CCR4-NOT, and decapping protein 2 (DCP2) [40,42]. The deadenylated and decapped mRNA target is then 5′-3′ degraded by exoribonuclease 1 (XRN1) [43]. Dysregulation of miRNA expression is correlated with many human diseases, including cancer [44,45]. miRNAs may act as either oncogenes or tumor suppressors under certain conditions, and their dysregulation has been shown to affect all the hallmarks of cancer (e.g., activation of proliferative signaling, invasion, and metastasis or inducing angiogenesis and drug resistance) [46]. A representation of miRNA biogenesis is reported in Figure 1.

## 4. Crosstalk between miRNA and DNA Methylation in Human Cancer

**Figure 2:**
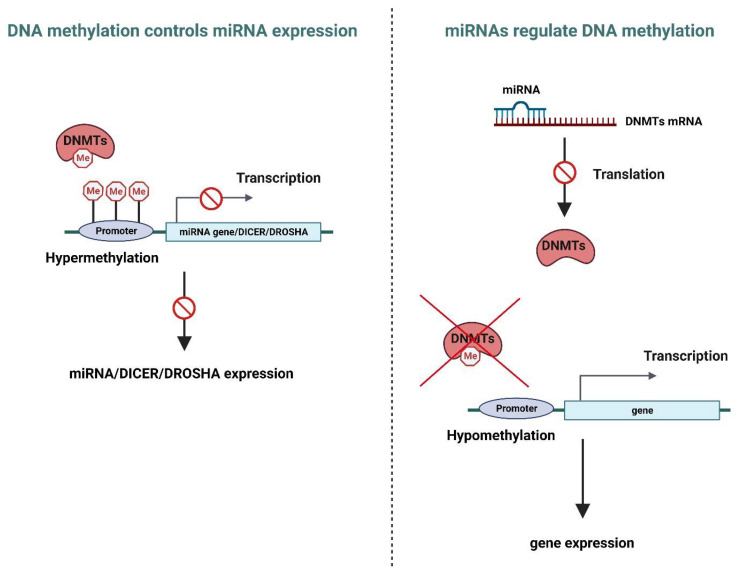
Mechanism of mutual regulation between miRNA and DNA methylation. Created with BioRender, https://www.biorender.com/ (accessed on 8 May 2023).

It is increasingly recognized that miRNA and DNA methylation can synergistically regulate transcription [47]. Increased interest in the crosstalk between miRNA expression and DNA methylation has fueled further investigations into how these regulatory mechanisms interact and impact each other. Such studies have uncovered how miRNAs can regulate DNA methylation by altering the expression of DNA methylases or their accessory proteins. Conversely, the methylation of the miRNAs’ promoter regions affects miRNAs’ expression [6,48] (Figure 2).

- Epigenetic control of miRNA expression (Figure 2, left)

Methylation of the miRNA promoter region regulates its expression. Several studies have shown that miRNA expression is reduced in response to promoter hypermethylation and has been observed in several human diseases [49]. Additionally, DNA methylation can indirectly impact miRNA expression through inhibition of the transcription of miRNA processing-related enzymes such as Dicer and Drosha [50].

- miRNA as regulators of DNA methylation (Figure 2, right)

MiRNAs modulate DNA methylation and interfere with the epigenetic machinery by altering the expression of DNA methylases or their accessory proteins. For example, the miR-29 family is known to target DNA methylases. Fabbri et al. determined that miR-29s have complementarity with the 3′UTRs of DNMT3A and DNMT3B and that their expression is inversely correlated with the expression of these enzymes in lung cancer [51].

The interaction between miRNA and DNA methylation has been extensively reviewed in the past years and has been observed in cancer and other pathologies [6,52,53,54]. Tao et al. recently reviewed how the interaction between miRNA and DNA methylation is determinant in atherosclerosis processes such as endothelial dysfunction, foam cell formation, and vascular smooth muscle cell proliferation [55]. In addition, in Alzheimer’s disease, miR-34a/b/c, miR-107, miR-124, miR125b, and miR-137, linked to the pathology progression, are epigenetically regulated [56]. In diabetic nephropathy, the mutual regulation between miRNA and the methylation machinery has been extensively reviewed by Sankrityayan et al. [57]. MiRNAs are also important regulators of DNA methylation in cardiovascular disease [58,59] and autoimmune diseases [60]. In systemic lupus erythematosus (SLE), the overexpression of miR-21 and miR-148a in CD4+ T cells contributes to DNA hypomethylation by repressing DNMT1 [61]. In rheumatoid arthritis, DNA methylation downregulates miR-34a* expression, promoting apoptosis resistance [62].

**Figure 3:**
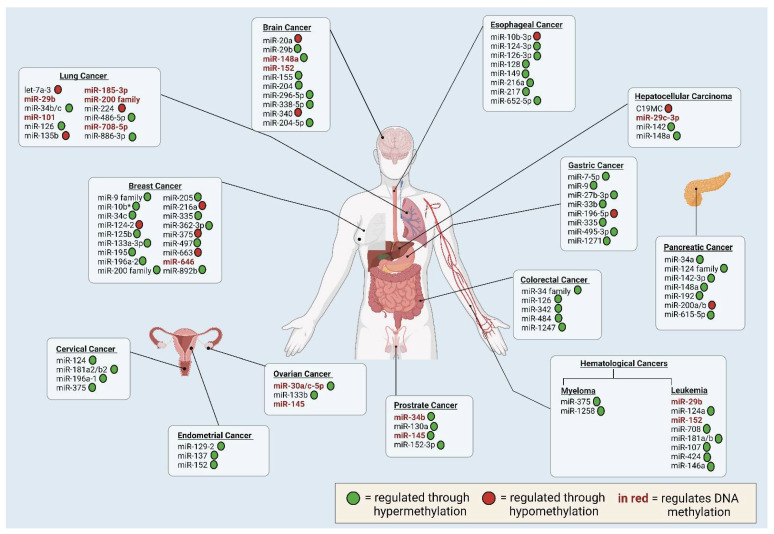
Overview of the epigenetically regulated miRNA and miRNA that regulates DNA methylation in various human cancers. Created with BioRender, https://www.biorender.com/ (accessed on 8 May 2023).

In the past years, many studies have focused on the mutual regulation between DNA methylation and miRNAs in cancer. In this section, we will explore this interaction in various human cancers (Figure 3), focusing on miRNAs that regulate DNA methylation (Table 1) and the effects of DNA methylation on miRNA expression (Table 2).

### 4.1. Lung Cancer

MiRNAs play an important role in lung cancer initiation and progression as they can act as oncogenes or tumor-suppressive genes [163]. As aforementioned, miRNA dysregulation in various human cancers, including lung cancer, has been linked to aberrant DNA methylation, a hallmark of human malignancies [164,165,166]. Thus, there is a need to explore the relationship between these epigenetic mechanisms to improve our understanding of lung tumorigenesis.

Recent publications have established that miRNAs can regulate DNA methylation directly or indirectly by modulating methylation regulators such as DNA methyltransferases (DNMTs) in lung cancer [76].

Liu et al. linked the ectopic expression of miR-708-5p with lowered luciferase activity in non-small-cell lung cancer (NSCLC) cells transduced with the *DNMT3a* wild-type coding DNA sequence (CDS) pGL3 vectors compared with cells transduced with mutated DNMT3a CDS, highlighting miR-708-5p’s direct influence on DNMT3a [67]. Investigators also found that enforced miR-708-5p expression decreased DNMT3a protein levels and consequently promoted CDH1 expression, a metastasis suppressor [67]. Interestingly, Li and colleagues demonstrated that radiation-mediated stimulation of miR-29b leads to reduced levels of DNMTs 1, 3a, and 3b and, subsequently, promoter hypomethylation and re-expression of *PTEN* [68]. MiR-101 is another miRNA that targets DNMT3a in lung cancer; its overexpression is associated with reduced DNMT3a levels, global DNA methylation, and ultimately, the re-expression of CDH1 [69]. DNMT3a has been identified as a target of the three miRNAs mentioned above, indicating that the DNMT3a-dependent DNA methylation is not regulated by a single miRNA but cooperatively by these three miRNAs and other unidentified miRNAs.

MiRNAs are also known to regulate DNA methylation by targeting important methylation-related proteins (MBPs), such as methyl CpG binding protein 2 (MeCP2), in various cancers, but knowledge of this epigenetic modulation of DNA methylation in lung cancer remains elusive [6]. Han and colleagues reported reduced MeCP2-WT luciferase activity after miR-185-3p transfection, suggesting that miR-185-3p negatively regulates MeCP2 in lung cancer [70]. Mechanistic studies investigating the epithelial-to-mesenchymal transition (EMT) suppressive function of MBD2, another crucial protein involved in the methylation process, demonstrated a positive correlation between MBD2 and the miR-200 family in lung adenocarcinoma [71]. This study shows that miR-200 can be targeted to regulate MBD2 in lung cancer [71]. These two studies show that miRNAs play an important role in regulating DNA methylation by targeting crucial proteins involved in the methylation process, but they also highlight the need for more studies to understand this epigenetic mechanism.

Hypermethylation of CpG islands of miRNA promoters is one of the most common epigenetic silencing mechanisms of tumor-suppressive miRNAs in lung cancer [167]. MiR-34b/c expression was restored after 5′-aza-DCR treatment in methylated small-cell lung cancer (SCLC) cell lines, potentially linking hypermethylation to the loss of miR-34b/c expression in SCLC [145]. Further functional analysis of miR-34b/c revealed that ectopic expression of the miR-34 family suppressed cell proliferation, invasion, and migration in SCLC cell lines [145]. Hypermethylation of miR-886-3p’s promoter suppresses its expression, leading to the downregulation of PLK1 and TGF-β1, inhibiting cell invasion, migration, and proliferation [144].

Over the past decade, researchers have also shown that miRNAs located in intronic regions of coding transcription units are often coordinately transcribed with their genes resulting in their co-regulation by DNA methylation [168]. Tessema and colleagues, utilizing combined bisulfite restriction analysis (COBRA), reported an inverse correlation between the hypermethylated *ANK1* promoter and intronic miR-486-5p expression levels in NSCLC cell lines [149]. Qualitative analysis using methylation-specific PCR revealed that *ANK1B* promoter hypermethylation could discriminate lung tumors by histology and smoking history. They found that lung adenocarcinomas (51%) had a higher *ANK1B* hypermethylation prevalence compared with squamous cell carcinoma (21%), and a similar trend was seen in cancer patients who were smokers (57%) compared with non-smokers (37%) [149]. This suggests the potential for *ANK1B* methylation as a diagnostic biomarker in lung cancer. DNA methylation of miR-126’s host gene, *EGFL7*, is linked to the repression of miR-126, which impedes cell invasion in NSCLC by targeting Crk, a key regulator of cell growth, motility, differentiation, and adhesion [150,151].

Oncogenic miRs, or “Onco-miRs,” are often upregulated in lung cancer, and hypomethylation is one of the processes implicated in their regulation [147,169]. Croce et al. demonstrated that miR-224 was significantly hypomethylated in NSCLC cell lines, and a positive correlation existed between high miR-224 levels and its promoter’s hypomethylation [146]. They also found that miR-224 promoted cell proliferation and migration by targeting TNFAIP1 and SMAD4 that are known for their respective proapoptotic and anti-migratory functions in lung cancer [146]. Let-7a-3 was reported to be significantly hypomethylated in lung adenocarcinomas compared with matched normal lung tissue samples [147]. Activation of let-7a-3 expression in vitro in NSCLC cell lines following 5-aza-2’-deoxycytidine (DAC) treatment revealed the involvement of DNA hypomethylation in the regulation of let-7a-3 in lung cancer [147]. Last, the investigators found that ectopic expression of let-7a-3 promoted anchorage-independent cell growth, thereby confirming this miR’s oncogenic function in lung cancer [147]. Upregulation of miR-135b in highly invasive CLI-5 lung cancer cells is observed due to promoter hypomethylation [148]. MiR-135b promotes cell invasion, tumor growth, and metastasis by targeting LZTS1 and some players of the Hippo signaling pathway [148].

### 4.2. Breast Cancer

Breast cancer (BC) is the leading cause of new cancer cases among American women [170]. The disease is histologically complex and can be characterized as hormone receptor-positive if the estrogen receptor (ER) and/or the progesterone receptor (PR) are expressed; these receptors and the human epidermal growth factor receptor 2 (HER2) are absent in triple-negative BC [171,172]. Studying the interplay between DNA methylation and miRNAs can provide insight into BC pathogenesis. In one of the earliest studies on DNA methylation and miRNAs in BC, COBRA was utilized to assess 61 miRNA gene candidates. Only miR-9-1 was significantly upregulated following treatment with 5-aza-2′-deoxycytidine [93]. Xenoestrogen repressed miR-9-3 in mammosphere-derived epithelial cells through an ERα-dependent mechanism that increased both H3K27me3 and H3K9me2 as well as hypermethylation of the miR-9-3 promoter [92]. The silencing of miR-9-3, which is involved in p53-related apoptotic pathways, enhances proliferation [92]. Mechanical compression caused by tumor growth in a restricted area can downregulate miR-9 through DNMT3A-dependent methylation of its promoter [94].

Further evaluation of DNA methylation data and genome-wide miRNA expression in the Oslo2 and The Cancer Genome Atlas Breast Invasive Carcinoma cohorts found 89,118 significant miRNA-CpG associations or miRNA-methylation quantitative trait loci (mimQTLs) composed of three miRNA clusters (immune, fibroblast, and estrogen signaling) and two CpG clusters [173]. In the invasive ductal carcinoma subtype of BC, upregulation of miR-646 promotes tumorigenesis by targeting TET1, impairing the demethylation of *IRX1* and consequently elevating HIST2H2BE [65]. On the other end, miR-646 has been shown to inhibit breast cancer cell growth and promoted cell death [174], showing a dual function of this miRNA in breast cancer that needs to be further evaluated. Additionally, the diminished expression of several regulatory miRNAs (miR-26a/b, miR-29a/b, and miR-148a/b) of DNMT3b is associated with aberrant DNA hypermethylation in the disease [175].

Several miRNAs have a reduction in expression attributable to hypermethylation of CpG islands within promoter regions, including miR-10b* [95], miR-892b [112], miR-133a-3p [99], miR-195 [100], miR-497 [100,109,110], miR-125b [98], miR-196a-2 [101], and miR-335 [106]. MiR-10b* targets BUB1, PLK1, and CCNA2, leading to a perturbation in cell proliferation in vitro and a reduction in tumor size in vivo [95]. MiR-892b targets components of the NFκB cascade (TRAB, TAK3, and TAB1) that promotes tumorigenesis [112]. MiR-133a-3p targets MAML1, thereby repressing migration and invasion [99]. MiR-195 targets Raf-1 and Ccnd1 and normally functions to inhibit colony formation and invasion [100]. MiR-497, in addition to targeting Raf-1 and Ccnd1, also represses GPRC5A and MUC1, contributing to chemotherapy resistance and malignancy, respectively [100,109,110]. MiR-125b targets ETS1, promoting cell cycle arrest and suppressing proliferation and tumorigenesis [98]. MiR-335 plays a role in inhibiting tumor reinitiation [106]. Methylation of *CLCN5* led to a reduction in miR-362-3p, which targets p130Cas, a regulator of receptor tyrosine kinase signaling [107]. Hypermethylation of the miR-34c promoter decreases its expression and further prevents the transcription factor Sp1 from binding to its regulatory element [96]. Conversion to stem-like/mesenchymal phenotype is associated with a loss of miR-200 family members [102]. One group found that miR-200a, miR-200b, and miR-429 are silenced by histone modifications, while miR-200c and miR-141 are repressed by DNA methylation [102]. Another study found that miR-200b is hypermethylated at two CpG islands [103]. MYC recruits DNMT3A to the promoter region of miR-200b, which catalyzes methylation of its CpG island, thereby reducing its expression and promoting EMT [66]. Although the tumor suppressor role of the miR-200 family is known, in some cases, miR-200 can have oncogene functions [176,177,178] and be upregulated in some breast cancer tissue compared with normal tissue [179].

Select miRNAs, including miR-375 [108], miR-663 [111], miR-216a [105], miR-205 [104], and miR-124-2 (B15), are upregulated by hypomethylation. Elevated levels of miR-375 in ERα-positive BC cells is a consequence of a loss in several epigenetic marks, including local DNA hypomethylation. MiR-375 enhances ERα signaling by targeting RASD1 [108]. There is a similar upregulation of hypomethylated miR-663 in chemoresistant BC cell lines and tumor tissues [111]. Interestingly, limonin attenuates stemness in BC by inducing hypomethylation of the miR-216a promoter, thereby increasing the levels of miR-216a, which targets WNT3A and represses the Wnt/β-catenin pathway [105]. Mel-18 induces the hypomethylation of CpG islands in the promoter region of miR-205 by impairing DNMT recruitment [104]. This increases miR-205 expression, which normally targets ZEB1 and ZEB2, leading to impaired EMT.

### 4.3. Brain Cancer

In brain cancers, several miRNAs are hypermethylated. In glioblastomas, miR-29b, miR-296-5p, and miR-204 have been found to be hypermethylated [79,82,84]. Hu and colleagues demonstrated that the long non-coding RNA DCST1-AS1 is upregulated in glioblastoma cells and induces the methylation of miR-29b. MiR-29b was shown to inhibit cell proliferation, and the effect is reversed when RNA DCST1-AS1 is overexpressed [79]. MiR-296-5p inhibits stem cell renewal by directly targeting HMGA1, which promotes the stem cell phenotype by altering the chromatin architecture of the stem cell maintenance transcription factor SOX2 [82]. This tumor suppressor role is in contrast with other findings that report miR-296-5p as invasion promoter in glioblastoma [180]. MiR-204 inhibits self-renewal, stem cell phenotype, and migration by the direct targeting of stemness-governing transcriptional factor SOX4 [84]. MiR-20a targets LRIG1, an inhibitor of receptor tyrosine kinases [85], and the demethylation of its promoter is positively correlated with temozolomide resistance.

In *IDHT*-mutated gliomas, miR-155 and miR-148a are hypermethylated; the hypermethylation of these two miRs was dependent on the *IDHT*-mutated status in gliomas [47,80]. MiR-148a directly targets DNMT1 [47], while miR-155 was shown to target FAM133A. The downregulation of FAM133A promotes cell migration and invasion. MiR-155 has also been recognized as an oncogene in glioma, contributing to tumor growth and progression [181], but more studies are needed to evaluate the context-dependent function of this miRNA.

In astrocytoma, miR-204-5p and miR-338-5p are hypermethylated [81,83]. MiR-204-5p inhibits cell migration and invasion by targeting Ezrin, whose expression is involved in late-stage tumor progression and metastasis [81,182], while miR-338-5p targets the pro-oncogene ETS-1 [83].

MiRNAs that target DNMT1 are dysregulated in brain cancers. MiR148a [47] and miR152-3p [63,64] directly target DNMT1 mRNA. MiR-152-3p expression is downregulated in glioblastoma cell lines; restoring the expression of miR-152-3p induced the demethylation of *NF2*, a known tumor suppressor [64]. In *IDH1* mutant glioblastomas, miR-148a is silenced by hypermethylation [47]. This hypermethylation increases the expression of DNMT1 and promotes the methylation of glioma-CpG island methylator phenotype (G-CIMP) genes such as *RBP1*, *CIDEB*, and *DLC1* [47].

A common treatment for neuroblastoma is all-trans-retinoic acid (ATRA), which induces the differentiation of neuroblastoma and decreases cell proliferation [183]. ATRA treatment results in the downregulation of DNMT1 and DNMT3B, resulting in the demethylation of various genes and miRNAs [63,78]. For example, ATRA treatment diminishes the expression of MYCN; MYCN is a repressor of miR-152 [63]. As mentioned above, miR-152 directly targets DNMT1 illustrating a regulatory network where ATRA treatment diminishes MYCN, miR-152 is expressed, and miR-152 downregulates DNMT1 [63]. Another example is the demethylation of miR-340 after ATRA treatment [78], which leads to overexpression of miR-340 and apoptosis or cell cycle arrest [78]. MiR-340 directly targets SOX2 mRNA, a transcription factor that maintains the undifferentiated state of stem and cancer cells [184].

### 4.4. Hematologic Cancers

Hematological malignancies are generally categorized into Hodgkin lymphoma, non-Hodgkin lymphoma, multiple myeloma, and leukemia. DNA methylation regulates the expression of miRNAs in leukemia and other hematological cancers.

With the intent of discovering aberrantly regulated miRNAs in chronic lymphocytic leukemia (CLL), Baer et al. profiled the epigenetic regulation of miRNAs in CLL compared with healthy B cells by the simultaneous detection of aberrant DNA methylation and miRNA promoters [185]. They found several miRNAs inversely correlating with DNA methylation and identified 12 miRNAs that were candidates for DNA methylation-dependent regulation. They validated miR-124-2, miR-129-2, miR-9-2, miR-551b, and miR-708, whose promoter showed consistent hypermethylation and reduced expression in an independent cohort of patients [185]. MiR-708 targets IKKβ, a kinase that induces NF-κB signaling, leading to the inhibition of the NF-κB pathway in chronic lymphocytic leukemia [87]. MiR-708 is known to behave both as an oncogene and a tumor suppressor in different cancers and several studies defines miR-708 as a contributor to leukemogenesis [186].

Garzon et al. demonstrated that miR-29b could reduce global DNA methylation in acute myeloid leukemia, thus inducing the re-expression of p15INK4b and *ESR1* via promoter DNA hypomethylation. MiR-29b directly targets DNMT3A and DNMT3B, repressing their translation. Moreover, miR-29b indirectly represses DNMT1 transcription by targeting its transactivator Sp1 [76].

In acute lymphoblastic leukemia (ALL), the promoter of miR-124a is hypermethylated, inducing its downregulation. As an effect, CDK6, a target of miR-124a, is overexpressed, leading to ALL cell growth through the CDK6-Rb oncogenic pathway [86].

Pallasch et al. identified miR-181a, miR-181b, miR-107, and miR-424 as hypermethylated and significantly downregulated in chronic lymphocytic leukemia (CLL). They all target the 3′UTR of the oncogene PLAG1, whose protein is upregulated in CLL cells compared with healthy donor B cells [88].

In myeloma, several tumor suppressor miRNAs, including miR-34b/c, miR-203, miR-129-2, and miR-342-3p, have been reported to be hypermethylated [187,188,189,190]. The tumor suppressor miR-1258, which targets PD-L1, is under transcriptional control of its host gene *ZNF385B*. This miRNA is silenced and methylated in a tumor-specific manner in myeloma, with an inverse correlation between methylation status and expression of *ZNF385B*/miR-1258 [90]. MiR-375 is significantly downregulated in CD138-positive plasma cells from multiple myeloma patients due to the hypermethylation of its promoter. Hence, the miR-375 overexpression inhibits the PDPK1, IGF1R, and JAK2 expression in human myeloma cell lines [91].

Several miRNAs have been epigenetically dysregulated in lymphomas, such as miR-203, miR-29a, and miR-9-1 [191]. In primary NK/T-cell lymphoma, promoter methylation and downregulation of miR-146a were observed [89]. MiR-146a is considered a tumor suppressor by repressing NF-kB signaling and thus inhibiting lymphoma cell proliferation and inducing apoptosis.

### 4.5. Gastrointestinal Cancers

#### 4.5.1. Esophageal Cancers

In esophageal squamous cell carcinoma (ESCC), CpG island promoter hypermethylation leads to reduced expression of exosomal miR-652-5p [120], miR-216a [124], miR-124-3p [125], and miR-149 [126]. Functionally, miR-652-5p targets PARG and VEGF to suppress cell growth and metastasis [120]. MiR-216a directly targets HMGB3 and decreases cell survival via the Wnt/β-catenin pathway [124]. MiR-124-3p targets EZH2, thereby inhibiting proliferation, migration, and invasion [125]. MiR-149 targets RNF2/Wnt/β-catenin axis and suppresses growth and metastases [126]. Zinc deficiency is associated with a high risk of esophageal cancers and induces pro-inflammatory COX-2 by suppressing its inhibitor, miR-128, through DNMT-mediated DNA methylation [122]. Exposure of esophageal adenocarcinoma cells to cigarette smoke condensate leads to DNMT3b-mediated hypermethylation of the miR-217 genomic locus, lowering levels of this miRNA that targets KLK7 [127]. As was observed in CRC, hypermethylation of *EGFL7* leads to a downregulation of miR-126-3p, which targets ADAM9 and subsequently reduces downstream signaling of the EGFR-AKT pathway [123]. Increased levels of miR-10b-3p in ESCC are attributed to the hypomethylation of promoter CpG sites [121]. This miRNA targets FOXO3 to induce cancer growth and metastasis [121].

#### 4.5.2. Gastric Cancer

In gastric cancer (GC), the hypermethylation of upstream CpG island(s) is linked to lower levels of miR-1271 [135], miR-9 [136], miR-33b [139], miR-27b-3p [140], miR-335 [141,142], and miR-495-3p [143]. MiR-1271 targets TEAD4, potentially leading to an enrichment of the YAP signature, and MAP2K1 (MEK1), thereby downregulating the MAPK/ERK pathway [135]. MiR-27b-3p targets GSTP1 and inhibits proliferation, migration, and invasion [140]. MiR-335 targets CRKL and RASA1, leading to repression in proliferation and migration while inducing apoptosis and cell cycle arrest [141,142]. MiR-495-3p regulates ten oncogenic epigenetic modifiers of HDAC2, KDM1A, KDM2B, KDM5B, CREBBP, EP300, MYST3, SMYD3, DNMT1, and MTA1 [143].

Hypomethylation of *HOXA10* is associated with higher levels of HOXA10 and miR-196b-5p in GC; the reconstitution of the TFF1, which acts as gastric tumor suppressor, induces methylation of HOXA10, thereby leading to decreased levels of HOXA10 and miR-196b-5p [137]. Gastric cancer stem cells (GCSCs) exhibit reduced miR-7-5p, partly mediated by DNA methylation of the miR-7-5p promoter [138]. When cultured with a methionine-deprived medium, the GCSCs have less promoter methylation and a significant increase in miR-7, which regulates sphere colony formation and invasion by targeting Smo and Hes1 [138].

#### 4.5.3. Hepatocellular Carcinoma

Hepatocellular carcinoma (HCC) accounts for approximately 90% of all primary liver cancers and is the third leading cause of cancer-related deaths worldwide [192]. Due to the lack of early-stage diagnostic markers, many HCC patients present clinically with late-stage disease, leading to a 5-year survival rate of less than 40% [161]. Thus, understanding genetic and epigenetic modifications in HCC is important to uncover potential diagnostic markers and therapeutic targets for HCC patients.

MiRNAs with tumor suppressive roles in HCC, such as miR-148a, are repressed due to CpG promoter hypermethylation [160]. Long and colleagues reported impaired HCC cell proliferation and cell cycle progression following enforced miR-148a expression [160]. A recent functional study demonstrated that the loss of miR-142 due to hypermethylation promoted cell viability, proliferation, and angiogenesis in HCC by upregulating TGF-β, a direct target of miR-142 [161]. In contrast, high expression levels of the chromosome 19 miRNA cluster (C19MC) due to promoter hypomethylation were observed in high T-stage HCC tumors with a high invasive ability [162]. Wu et al. demonstrated that miR-29c-3p regulates DNA methylation by targeting DNMT-3B leading to the methylation of large tumor suppressor gene 1 (*LATS1*), which inactivates the Hippo signaling pathway [77]. Hippo signaling pathway is known for its oncosuppressive role of inhibiting HCC cell proliferation and promoting apoptosis [193].

#### 4.5.4. Pancreatic Cancer

Pancreatic cancer is a highly lethal cancer with high rates of metastases and poor prognosis [194]. The most common subtype of pancreatic cancer, pancreatic ductal adenocarcinoma (PDAC), accounts for 90% of pancreatic cancer cases [195]. Aberrant DNA methylation of miRNAs has been reported in PDAC [196], with upregulation of DNMT1 commonly found in PDAC, particularly in *TP53*-mutant PDAC cells [130]. Several miRNAs, such as miR-192, miR-615-5p, miR-142-3p, miR-148, miR-34a, the miR-200 family, and the miR-124 family, are dysregulated in their DNA methylation in pancreatic cancer. Hypermethylation of miR-192 upregulates Vimentin, an EMT marker, promoting metastasis [128]. MiR-192 directly targets SERPINE1 mRNA. Serpine1 is known to regulate cell proliferation and invasion. MiR-615-5p is hypermethylated in PDAC [129]. It targets IFG2, which promotes cell proliferation, invasion, and migration. miR-142-3p was observed to be hypermethylated in *TP53*-mutant PDAC cells, and its hypermethylation is dependent on DMNT1 expression [130]. MiR-148 is hypermethylated in PDAC [197], and its restoration downregulates the Wnt/β-catenin signaling pathway and suppresses cell proliferation [131]. MiR-34a targets the Notch signaling pathway. When miR-34a is hypermethylated, Notch signaling is activated and promotes drug resistance in PDAC cells [133]. The miR-124 family is hypermethylated in PDAC. The miR-124 family inhibits cell proliferation and metastasis by targeting Rac1 [134], a pro-tumor enhancer that activates the MKK4-JNK-c-Jun pathway. The miR-200 family is often upregulated in pancreatic cell lines and primary tumor tissues, which reflects an upregulation of miR-200a and 200b in serum of pancreatic cancer patients. This upregulation is due to the hypomethylation of the miR-200 promoter region [132]. Nevertheless, the miR-200 family is known to act as a tumor suppressor and mediator of EMT in pancreatic cancer [198]. The miR-200 upregulation in this cancer can be related to the hypomethylation of its promoter; however, no defined effects on EMT were observed after miR-200 treatments [132]. The controversy surrounding miR-200 upregulation in tumors with its tumor suppressor function has yet to be fully understood.

#### 4.5.5. Colorectal Cancers

Concurrent downregulation of miR-342 and *EVL*, its host gene, is attributable to methylation of the *EVL*/miR-342 locus [113]. The epigenetic silencing of miR-342 can induce anti-apoptotic pathways [113]. Similarly, the methylation of *EGFL7* leads to reduced miR-126, a tumor suppressor that targets VEGF and induces anti-angiogenic effects in CRC [119]. Several groups [114,115,116] have reported hypermethylation of the CpG island in the miR-34b/c promoter in CRC cell lines and primary tumor tissues and fecal specimens. The re-expression of miR-34b/c, which targets MET, CDK4, and SFRS2, markedly reduces colony formation in tested CRC cell lines [114]. Increased methylation of miR-34a was observed in CRC tumors with liver metastasis and has potential prognostic value for distant metastases when combined with elevated c-Met and β-catenin expression [116]. In hypermethylated CRC tissues and cell lines, miR-1247 expression is reduced, and its target, MYCPB2, is elevated [117]. Functionally, miR-1247 has tumor-suppressive capacities by impairing cell viability and inducing apoptosis [117]. In CRC with microsatellite instability, there are lower levels of miR-484 due to CpG island promoter methylation [118]. This miRNA targets CD137, which was linked to halting IL-8 production [118].

### 4.6. Gynecological Cancers

#### 4.6.1. Cervical cancer

DNA methylation of miR-124 was found in colon, breast, lung cancer, leukemia, and lymphoma [199]. Wilting et al. evaluated the role of DNA methylation-based silencing of miR-124 during cervical carcinogenesis. They showed that all three loci encoding the mature miR-124 (miR-124-1/-2/-3) were methylated in cervical cancer cell lines. As a result, the expression of mature miR-124, which has tumor suppressor activity in cervical cancer, was reduced [152]. A recent study demonstrated that the promoters of miR-375 and miR-196a-1 are hypermethylated in squamous cell carcinoma tissue compared with the normal cervical epithelium and cervical intra-epithelial neoplasia, leading to their downregulation at transcript levels. In vitro studies showed that the downregulation of miR-375 and miR-196a-1 inhibit the proliferation of SiHa cells, revealing a possible tumor suppressor role of these miRNAs in cervical cancer [153]. By contrast, Shen et al. observed that the overexpression of miR-375 in cervical cancer cells decreased sensitivity to paclitaxel in vitro and in vivo [200], raising additional questions regarding the dual function of miRNAs that need to be answered. In addition, the under expression of miR-181a2/181b2 is detected in over 45% of cervical cancers and is partially induced by the hypermethylation of its promoter region. MiR-181a2/181b2 exerts tumor suppressor effects in vitro and in vivo through targeting of PIK3R3/Akt/FoxO signaling, and its reduction is associated with poor prognosis and advanced stage cervical cancer [154].

#### 4.6.2. Ovarian Cancer

In ovarian cancer, Han and colleagues have reported an interesting feedback loop between miR-30a/c-5p and DNMT1 [72]. The researchers showed that levels of miR-30a/c-5p were drastically reduced in cisplatin-resistant ovarian cancer cells (CP70) compared with cisplatin-sensitive cells (A2780). This reduction was induced by the increased methylation levels in the promoter regions of miR-30a/c-5p precursor genes and a higher level of DNMT1 maintenance in CP70 cells compared with A2780 cells. Functional studies revealed that miR-30a/c-5p could attenuate cisplatin resistance and EMT by targeting Snail. By contrast, the overexpression of DNMT1 promotes cisplatin resistance and partial EMT in ovarian cancer cells. Interestingly, this group found that miR30a/c-5p directly targets DNMT1 3′UTR, inhibiting its expression and directing a feedback loop that uncovers additional mechanisms in ovarian cancer drug resistance.

In addition, miR-145 and miR-133b were downregulated in ovarian cancer tissue and serum from ovarian cancer patients, where miR-145 indirectly promotes miR-133b expression. Mechanistically, miR-133b targets PKM2, inhibiting the Warburg effect in ovarian cancer, and miR-145 can inhibit the recruitment of DNMT3A in the promoter of miR-133b by targeting c-myc, thereby promoting miR-133b expression [73].

#### 4.6.3. Endometrial Cancer

Several miRNAs are epigenetically downregulated in endometrial cancer. Tsuruta and colleagues identified miR-152 as tumor suppressor miRNA silenced by DNA hypermethylation in endometrial cancer. miR-152 fulfills its tumor suppressor activity by targeting E2F3, MET, and Rictor [155]. MiR-137, which targets EZH2 and LSD1 and inhibits tumor growth, is frequently hypermethylated and repressed in endometrial cancer [156]. Hypermethylation of the miR-129-2 CpG island was observed in endometrial cancer and was associated with the concomitant gain of SOX4 expression, an oncogene target of miR-129-2 [157]. The overexpression of SOX4 can partially be caused by the epigenetic repression of miR-129-2.

### 4.7. Prostate Cancer

Prostate cancer accounts for 27% of cancer diagnoses in men [201]. With the intent of discovering new epigenetically regulated miRNA loci in prostate cancer, Jerónimo’s group identified miR-152-3p with decreased expression associated with promoter hypermethylation in prostate cancer tissues [158]. The same group previously found miRNAs globally downregulated in prostate cancer cell lines, an effect reversed by treatment with 5-Aza-CdR, a demethylating agent. Among the deregulated miRNAs, they found miR-130a downregulated and hypermethylated in prostate cancer tissue compared with morphological normal prostate tissue. MiR-130a overexpression inhibited cell viability, increased apoptosis, and reduced the invasive potential of prostate cancer cell lines [159].

Majid et al. reported that the downregulation of miR-34b in prostate cancer tissues and cell lines is driven by the hypermethylation of its promoter [74]. Remarkably, the exogenous overexpression of miR-34b in PC3 and LNCaP cells induces the downregulation of DNMT1 and DNMT3b, with DNMT1 as a direct target of miR-34b. A decrease in cell proliferation inhibited EMT, and induced apoptosis was observed in PC3 and LNCaP cells overexpressing miR-34b. It has been reported that DNMT3b is upregulated and miR-145 is downregulated in prostate cancer cells [202,203], and either the repression of DNMT3b or the overexpression of miR-145 can suppress the proliferation and migration of PC3 cells [204,205]. Xue et al. demonstrated that DNMT3b is a direct target of miR-145, whose transcription is controlled by DNMT3b-induced methylation, proposing crosstalk between these two epigenetic factors in prostate cancer [75]. Additionally, they showed that the overexpression of miR-145 and the downregulation of DNMT3b sensitizes PC3 cells to irradiation.

## 5. The Use of Epigenetics as Biomarkers

The aberrant expression of miRNAs has been the subject of intense investigation for years in cancer research. MiRNAs can be easily detected in tissues but also in circulation by non-invasive liquid biopsy [206,207]. Their stability and easy detection render miRNA a suitable biomarker for human cancer diagnosis, prognosis, and therapeutics [208,209]. Furthermore, many studies are ongoing to propose miRNA-based cancer therapies [210,211,212]. Liquid biopsy is increasingly being used for helping cancer diagnosis thanks to several advantages compared with the conventional biopsy, such as minimal invasiveness, pain, and risk of complication [213]. Circulating tumor-related miRNAs can be found in several bodily fluids and be used for cancer screening, diagnostics, and prognostics [213]. These miRNAs are intensively studied in several cancer types and are easily detectable in serum [214,215,216] and plasma [217,218,219].

Other biological fluids can be also informative. In urine samples, it is possible to detect miRNAs with diagnostic and prognostic biomarker in esophageal [220], cervical [221], bladder [222,223,224], colorectal [225], and prostate cancers [226].

The aberrant expression of miRNAs can be found in cerebrospinal fluid, pancreatic juice, sputum, and pleural effusion and could be used as potential biomarkers for brain [227], pancreatic [228], and lung cancers [229,230], respectively.

DNA methylation profiles of miRNAs can be used as a signature to define tumor type, clinical prognosis, and treatment response [74,231,232]. Indeed, dysregulation of DNA methylation is likewise ubiquitous across various cancer types and is considered a hallmark of cancer [233,234,235]. Disease- and exposure-related methylation changes are detectable in blood, potentially allowing them to serve as biomarkers for cancer and the immune response [236,237,238].

Over the last several years, the number of studies reporting the utility of DNA methylation as biomarkers have vastly increased [239]. One or more methylation sites were observed in promoters of miRNAs or their associated enzymes, and the presence of methyl groups can be used as a biomarker for tumor incidence and prevalence [240]. For example, frequent methylation of miR-124a, miR-34b/c, miR-9-1, miR-9-2 and miR-9-3, miR-10b, miR-203, miR-196b, and miR-132/212 has been found in acute lymphoblastic leukemia patients [241]. Importantly, patients with non-methylated miRNA promoters also had lower mortality and higher overall survival rates compared with patients with methylated miRNA promoters [241]. Su et al. analyzed the sputum of 117 early-stage NSCLC patients compared with 174 cancer-free smokers. They integrated the expression levels of miR-31 and miR-210 and methylation levels of genes *RASSF1A* and *3OST2* and validated them in a second cohort of patients. The panel of biomarkers yielded high sensitivity (87.3%) and specificity (90.3%) for early detection of NSCLC [229]. Heller et al. investigated the methylation status of miRNAs in primary tumor samples and corresponding non-malignant lung tissue samples of NSCLC patients. Methylated DNA immunoprecipitation followed by custom-designed tiling microarray analyses (MeDIP-chip) found that miR-10b, miR-1179, miR-137, miR-572, miR-3150b, and miR-129-2 were significantly upregulated in tumor tissue compared with non-malignant tissue. The loss of miR-889-3p due to its promoter hypermethylation in SCLC tumors was associated with significantly shorter overall survival, progression-free survival, and distance metastasis-free survival, highlighting the potential use of miR-889-3p as a prognostic biomarker in SCLC [144]. In NSCLC, the miR-129-2 gene is more frequently methylated in stage III than in stage I/II patients [242], and the same miRNA is associated with shorter disease-free survival of prostate cancer patients [243], as well as shorter overall survival and disease-free survival of hepatocellular carcinoma patients [244]. Another study reported a biomarker of serum miR-24 and miR-30c expression combined with *CRIP3* methylation in urine samples useful to monitor prostate cancer patients on active surveillance [245]. In malignant prostatic tissues, the promoter methylation levels of miR-34b/c, miR-129-2, miR-152, miR-193b, miR-663a, and miR-1258 were significantly higher than in morphologically normal prostate tissue. In addition, promoter methylation levels of miR-34b/c, miR-663a, and miR-1258 were associated with higher pathological stages [246]. Interestingly, the same group tested the methylation level of miR-34b/c, miR-193b, and miR-1258 in urine samples and found that miR-193b performed best as a biomarker for prostate cancer, with AUC = 0.96, sensitivity = 91.6%, and specificity = 95.7%.

In astrocytoma, the methylation status of miR-338-5p was shown to increase with stage and correlated with disease severity [83]. In cervical cancer, the promoter hypermethylation of miR-124-2, *SOX1*, *TERT*, and *LMX1A* genes directly correlated with the presence of grade 2 cervical intraepithelial neoplasia, and the methylation of miR-124-2 represents a promising biomarker for precursor lesions with sensitivity = 86.7% and specificity = 61.3% [247]. In addition, a 14-year follow-up post hoc analysis on the POBASCAM trial has recently shown that a negative *FAM19A4*/mir124-2 methylation test provides a low cervical cancer risk in HPV-positive women of 30 years and older [248]. Additionally, in breast cancer, young women are generally diagnosed at advanced stages of the disease and were found to have a significant overexpression of hypomethylated miR-124-2, which is associated with poor survival [97]. In primary hepatocellular carcinoma specimens, miR-1-1 was the first miRNA reported to be targeted by aberrant DNA methylation and downregulation compared with matching normal liver tissues [249].

A comprehensive study performed on patients with gastric cancer reported the upregulation of miR-106a in cancer tissue compared with the normal adjacent tissue, partially due to the hypomethylation of its promoter. Additionally, miR-106a was upregulated in the plasma of gastric cancer patients compared with healthy controls, and its expression was downregulated after gastrectomy, highlighting the relevance of this miRNA as a diagnostic marker [250].

## 6. Conclusions

The underlying mechanisms driving the aberrant expression of miRNAs remain the focus of intense research. The epigenetic deregulation of miRNAs is a new area of investigation that is drawing interest and is increasingly being studied in relation to cancer.

Aberrant expression of miRNAs is observed across a variety of human cancers, and it is partially explained by epigenetic factors, adding a level of complexity to the regulation of these small molecules. We focused on the interplay between DNA methylation and miRNAs, specifically on how the methylation of miRNAs promoters contributes to their deregulation. From a translational point of view, the expression of miRNAs can be evaluated in tumor tissues to evaluate the tumor burden and potentially inform cancer diagnosis clinically. Additionally, considering the stability and easy detection of miRNAs in bodily fluids, their expression could be used for helping to screen and diagnose human cancers.

The methylation levels of the miRNA promoter can be a mechanism that leads to the aberrant expression of miRNAs in cancer, and it should be considered to improve our understanding of tumor pathogenesis and progression. Ultimately, to have a functional consequence in cancer, the mechanisms behind this regulation could be used as targeted therapy for cancer. The hypermethylation of tumor suppressor genes and miRNAs is reported in several tumors and could potentially be targeted using inhibitors of DNA methylation to restore tumor suppressor activities. Several inhibitors of DNA methylation are currently used in clinical trials and have effects on solid tumors [251,252].

Although relatively young, the interest in this new field is increasing. It is important to consider the epigenetic regulation of miRNAs to expand our knowledge of cancer pathogenesis and potential therapeutic strategies.

## Figures and Tables

**Table 1:** microRNAs that regulate DNA methylation in cancer.

Cancer	miRNA	Effect on DNA Methylation	Functional Consequence	Ref
Brain	miR-152	DNMT1	Negatively affects cell invasiveness. miR-152 is upregulated when treating neuroblastoma with ATRA and can be a possible marker for treatment effectiveness.	[63]
	miR-148a	DNMT1	miR-148a directly targets DNMT1 in *IDHMT* gliomas, thereby altering the DNA methylation status of other miRNAs.	[47]
	miR-152-3p	DNMT1	miR-152-3p is proposed to target DNMT1, which targets *NF2*. DNMT1 methylates and downregulates miR-152-3p. Overexpression of both NF2 and miR-152-3p induces apoptosis.	[64]
Breast	miR-646	Targets TET1	It affects DNA demethylation and leads to a downregulation of IRX1, which normally suppresses HIST2H2BE. This promotes malignancy and tumorigenesis in breast cancer’s invasive ductal carcinoma subtype.	[65]
	miR-200b	Targets DNMT3A	A potential feedback loop exists where miR-200b targets DNMT3A yet is hypermethylated through MYC-recruited DNMT3A.	[66]
Lung	miR-708-5p	Regulates DNMT3a	Upregulation of miR-708-5p is associated with decreased DNMT3a protein levels, leading to elevated levels of CDH1, a metastasis suppressor.	[67]
	miR-29b	Regulates DNMT1, 3a, and 3b	High miR-29b levels lead to reduced levels of DNMTs 1, 3a, and 3b. Subsequently, *PTEN’s* promoter is hypomethylated and re-expressed, resulting in tumor growth delay.	[68]
	miR-101	Regulates DNMT3a	Overexpression of miR-101 is associated with reduced DNMT3a levels and global DNA methylation and, ultimately, the re-expression of CDH1, a tumor suppressor.	[69]
	miR-185-3p	Regulates MeCP2	Reduced MeCP2-WT luciferase activity was reported after ectopic miR-185-3p expression, suggesting that miR-185-3p negatively regulates MeCP2, a methylation-related protein, in lung cancer.	[70]
	miR-200 family	Regulates MBD2	A positive correlation between MBD2 and the miR-200 family in lung adenocarcinoma clinical samples has been reported and suggests miR-200 as a potential target for modulating MBD2, a protein involved in the methylation process.	[71]
Ovarian	miR-30a/c-5p	DNMT1	Directly targets DNMT1 and negatively regulates cisplatin resistance and EMT.	[72]
	miR-145	DNMT3A	Indirectly inhibits DNMT3A by targeting c-myc.	[73]
Prostate	miR-34b	DNMT1 and DNMT3b	Reduces DNMT1 and DNMT3b, decreases cell proliferation, inhibits EMT, and induces apoptosis in PC3 and LNCaP cells.	[74]
	miR-145	DNMT3b	Targets DNMT3b. Its overexpression sensitizes PC3 cells to irradiation.	[75]
Leukemia	miR-29b	DNMT1, DNMT3A, DNMT3B	Directly targets DNMT3A and DNMT3B and indirectly downregulates DNMT1 by targeting its transactivator SP1.	[76]
Hepatocellular	miR-29c-3p	DNMT-3B	Leads to the methylation of *LATS1*, which inactivates the Hippo signaling pathway.	[77]

**Table 2:** DNA methylation regulating miRNAs in cancer.

Cancer	miRNA	Mechanism	Functional Consequence	Ref
Brain	miR-340	Demethylated with ATRA treatment	Targets SOX2 transcription factor, which is responsible for maintaining stem cells undifferentiated.	[78]
	miR-29b	Hypermethylation	Overexpression of DCST1-AS1 induces the methylation and subsequent downregulation of miR-29b, which normally inhibits cell proliferation.	[79]
	miR-155	Hypermethylation	Targets FAM133A, a negative regulator of cell invasion/migration, by regulating MMP14.	[80]
	miR-204-5p	Hypermethylation	Targets Ezrin and inhibits invasion/migration.	[81]
	miR-148a	Hypermethylated	DNMT1 hypermethylates miR-148a, which itself directly targets DNMT1. The suppression of miR-148a is linked to DNA methylation changes.	[47]
	miR-296-5p	Hypermethylation	Inhibits stem cell self-renewal by targeting HMGA1, a chromatin remodeling protein that regulates the stem cell transcription factor SOX2.	[82]
	miR-338-5p	Hypermethylation	Targets the protooncogene EST-1, which is associated with proliferation and invasion of cancer cells.	[83]
	miR-204	Hypermethylation	Targets SOX4 and EphB2 and reduces cell invasion and tumorigenesis. It is downregulated in glioblastoma by hypermethylation.	[84]
	miR-20a	Demethylated	DNMT1 methylates and downregulates miR-20a, which normally targets LRIG1; this leads to chemosensitivity.	[85]
Leukemia	miR-124a	Hypermethylation	miR-124a is hypermethylated and downregulated in ALL, leading to cell growth through the CDK6-Rb oncogenic pathway.	[86]
	miR-708	Hypermethylation	miR-708 targets IKKβ and regulates the NF-κB pathway.	[87]
	miR-181a/b, miR-107, miR-424	Hypermethylation	Target 3′UTR of the oncogene PLAG1.	[88]
	miR-146a	Hypermethylation	Represses NF-KB signaling.	[89]
Myeloma	miR-1258	Hypermethylation	Targets PDL1.	[90]
	miR-375	Hypermethylation	Represses the expression of PDPK1, IGF1R, and JAK2 in HMCLs.	[91]
Breast	miR-9 family	Increased H3K27me3 and H3K9me2 along with hypermethylation of the miR-9-3 promoter CpG island	Results in a downregulation of miR-9-3, which is involved in p53-related apoptotic pathways.	[92]
		Hypermethylation	Function was not assessed, but there is a statistical significance in miR-9-1 methylation status in breast carcinoma vs benign tumors.	[93]
		Mechanical compression induces DNMT3A-mediated hypermethylation of promoter	Results in a downregulation of miR-9 and an upregulation of its targets (LAMC2, ITGA6, EIF4E), leading to the production of vascular endothelial growth factors.	[94]
	miR-10b* (Note: * refers to an old miRNA nomenclature)	Hypermethylation of two CpG islands upstream of miR-10b/10b* locus	Results in a downregulation of miR-10b*, which was demonstrated to inhibit cell proliferation in vitro and tumor growth in vivo. The targets of miR-10b * include BUB1, PLK1, and CCNA2.	[95]
	miR-34c	Hypermethylation	Reduces miR-34c in breast tumor-initiating cells, which normally targets Notch4, reducing migratory ability and EMT. The hypermethylation of the miR-34c promoter prevents the transcription factor Sp1 from binding to its regulatory element.	[96]
	miR-124-2	Hypomethylation	Overexpresses miR-124-2, particularly in young women with breast cancer, and is associated with poor survival in patients.	[97]
	miR-125b	Hypermethylation	Reduces miR-125b, which normally targets ETS1, promoting cell cycle arrest and suppression of proliferation and tumorigenesis.	[98]
	miR-133a-3p	Hypermethylation	Reduces tumor suppressor miR-133a-3p, leading to an increase in its target MAML1, thereby promoting metastasis, proliferation, invasion, and stemness. A feedback loop exists where MAML1 upregulates DNMT3A, leading to hypermethylation of the promoter of miR-133a-3p.	[99]
	miR-195	Hypermethylation of select upstream CpG islands to promoter	Leads to a downregulation of miR-195 and upregulation of its targets Raf-1 and Ccnd1. miR-195 normally functions to inhibit colony formation and invasion.	[100]
	miR-196a-2	Hypermethylation of CpG island upstream of the miR-196a-2 precursor	The effect of CpG island hypermethylation on mature miR-196a-2 levels was not confirmed, but the increased methylation of this site is correlated with increased breast cancer risk.	[101]
	miR-200 family members	Hypermethylation	Reduces miR-200c and miR-141 levels, thereby leading to stem-like/mesenchymal phenotype.	[102]
		Hypermethylation of two CpG cites denoted “P1” and “P2”	Reduces miR-200b. P1 hypermethylation is associated with metastatic lymph node samples, while P2 hypermethylation is associated with estrogen or progesterone receptor loss, and its hypomethylation is associated with HER2 and androgen receptor expression.	[103]
		MYC recruits DNMT3A and induces hypermethylation of CpG island to promoter	Reduces miR-200b in triple negative breast cancer, leading to EMT. miR-200b was demonstrated to target DNMT3A, suggesting a regulatory feedback loop.	[66]
	miR-205	Hypermethylation of CpG sites in the promoter region since DMNT recruitment is no longer inhibited by Mel-18	Leads to a downregulation of miR-205 and increased level of its targets ZEB1 and ZEB2, which promote epithelial-to-mesenchymal transition.	[104]
	miR-216a	Limonin mediates hypomethylation of CpG island in its promoter	Increases levels of miR-216a, which targets WNT3A, inactivating the Wnt/β-catenin signaling cascade and attenuating stemness and adriamycin resistance.	[105]
	miR-335	Genetic copy loss at the miR-335 locus along with hypermethylation of CpG island in the promoter of miR-335/Mest	Reduces levels of the tumor suppressor miR-335, which has a role in suppressing tumor reinitiation, invasion, and metastasis.	[106]
	miR-362-3p	Hypermethylation of *CLCN5* promoter	Leads to a reduction in tumor suppressive miR-362-3p, which targets p130Cas, a regulator of receptor tyrosine kinase signaling, and normally suppresses cell viability, migration, invasiveness, and tumor growth.	[107]
	miR-375	Hypomethylation	Contributes to an upregulation of miR-375, which targets RASD1 and leads to enhanced ERα signaling and cell proliferation.	[108]
	miR-497	DNMT-mediated methylation of CpG islands to promoter	Leads to a downregulation of miR-497 and upregulation of its targets Raf-1, Ccnd1, GPRC5A, and MUC1. miR-497 normally inhibits colony formation, invasion, and malignancy and promotes apoptosis. The repression of miR-497 is linked to chemotherapy resistance and metastases.	[100,109,110]
	miR-663	Hypomethylation of CpG sites	Leads to an upregulation of miR-663, which targets HSPG2 in multidrug-resistant breast cancer cell lines and leads to chemoresistance.	[111]
	miR-892b	Hypermethylation	Leads to a reduction in miR-892b, which normally suppresses several components of the NFκB cascade, including TRAF, TAB3, and TAK1, and decreases tumor growth, metastasis, and angiogenesis in breast cancer cells.	[112]
Colorectal	miR-342	Methylation of the *EVL*/miR-342 locus	Resulted in a downregulation of miR-342, potentially inducing anti-apoptotic pathways.	[113]
	miR-34b/c	Hypermethylation of neighboring CpG island	Resulted in the epigenetic silencing of the tumor suppressors miR-34b/c, whose functions include suppressing colony formation.	[114,115]
	miR-34a	Increased methylation of CpG islands in the promoter and transcribed region	Is elevated in primary tumors with liver metastases. When combined with elevated c-Met and β-catenin expression, it has potential prognostic value for distant metastasis.	[116]
	miR-1247	Hypermethylation of promoter regions	Leads to decreased levels of miR-1247 in hypermethylated CRC cell lines and tissue specimens, leading to an upregulation of its target MYCBP2. Introduction of miR-1247 impairs cell viability, induces apoptosis, and inhibits cell motility in vitro while reducing tumor mass and size in vivo.	[117]
	miR-484	Hypermethylation of CpG on the island promoter	Is observed in CRC with microsatellite instability, leading to lower levels of miR-484, which functions as a tumor suppressor and targets CD137L, arresting IL-8 production.	[118]
	miR-126	Methylation of its host gene *EGFL7*	Leads to the silencing of miR-126, which targets VEGF and acts as a tumor suppressor by inhibiting cell growth, invasion, migration, and angiogenesis.	[119]
Esophageal	miR-652-5p	Hypermethylation	Reduces the expression of exosomal miR-652-5p, which targets PARG and VEGF, suppressing cell growth and metastasis in vitro and in vivo.	[120]
	miR-10b-3p	Hypomethylation of CpG islands upstream to the miR-10-3p gene	Increases the expression of miR-10b-3p, which targets FOXO3, inducing cell growth and metastasis in vitro and in vivo.	[121]
	miR-128	Hypermethylation	In response to zinc deficiency, there are increased levels of DNMT1 and DNMT3B. The methylation of miR-128 leads to an upregulation of its target: the pro-inflammatory COX-2.	[122]
	miR-126-3p	Hypermethylation of its host gene *EGFL7*	Resulted in a downregulation of miR-126-3p, which suppresses proliferation and migration. It targets ADAM9 and subsequently reduces the downstream signaling of the EGFR-AKT pathway.	[123]
	miR-216a	Hypermethylation	Resulted in a downregulation of miR-216a, which targets HMGB3 and decreases cell survival through the Wnt/β-catenin pathway.	[124]
	miR-124-3p	Hypermethylation of miR-124 loci	Reduces levels of miR-124, which inhibits proliferation, migration, and invasion by targeting EZH2.	[125]
	miR-149	Hypermethylation	Leads to low expression of miR-149, which targets RNF2, impacting the Wnt/β-catenin pathway and suppressing growth and metastases.	[126]
	miR-217	Cigarette smoke condensate induces DNMT2b-dependent hypermethylation of the miR-217 genomic locus.	Leads to reduced levels of miR-217, which targets KLK7 and decreases proliferation and invasion.	[127]
Pancreatic	miR-192	Hypermethylation	Low levels of miR-192 promote EMT, while its overexpression inhibits cell migration and invasion. Specifically, miR-192 affects the expression of SERPINE1.	[128]
	miR-615-5p	Hypermethylation	miR-615-5p reduces cell proliferation, migration, invasion, and tumor growth in vivo. It directly targets IGF2, which is responsible for the cancerous phenotype. Rescue of IGF2 expression impairs the tumor suppressive activity of miR-615-5p.	[129]
	miR-142-3p	Hypermethylation	DNMT1 is upregulated with p53 mutant pancreatic ductal adenocarcinoma and methylates miR-142-3p in a p53 mutation-dependent manner. The overexpression of miR-142-3p inhibits cell invasion in vitro.	[130]
	miR-148a	Hypermethylation	miR-148a is methylated in pancreatic cancer. Restoration of miR-148 downregulates the Wnt/β-catenin pathway and inhibits mesenchymal-to-epithelial transition.	[131]
	miR-200a/b	Hypomethylation	miR-200 is primarily hypomethylated in pancreatic cancers, which contributes to its upregulation.	[132]
	miR-34a	Hypermethylation	miR-34a is methylated by DNMT1, leading to the activation of the Notch pathway, which promotes drug resistance.	[133]
	miR-124 family (124-1/2/3)	Hypermethylation	miR-124 inhibits cell proliferation and metastasis by targeting Rac1, a pro-tumor enhancer that activates the MKK4-JNK-c-Jun pathway.	[134]
Gastric	miR-1271	Hypermethylation	Leads to lowered levels of miR-1271, which targets TEAD4, potentially leading to an enrichment of the YAP signature and represses MAP2K1 (MEK1), thereby downregulating the ERK/MAPK pathway.	[135]
	miR-9	Hypermethylation of promoter-proximal CpG island	Results in a downregulation of miR-9, which is associated with the clinicopathological features of tumor size and lymph node metastasis.	[136]
	miR-196-5p	Hypomethylation of *HOXA10* promoter	Is associated with increased levels of HOXA10 and miR-196-5p, thereby enhancing proliferation and invasion. TFF1 reconstitution represses HOXA10 and miR-196-5p by inducing methylation of *HOXA10*.	[137]
	miR-7-5p	Hypermethylation	Partially mediates the lower expression of miR-7-5p in stem cells. When cultured with methionine-depleted medium, there is less methylation of the promoter and greater expression of miR-7-5p, which regulates colony formation and cell invasion by targeting Smo and Hes1.	[138]
	miR-33b	Hypermethylation	Downregulates miR-33b, which suppresses proliferation, migration, and invasion, possibly by regulating c-Myc.	[139]
	miR-27b-3p	Hypermethylation	Leads to decreased miR-27b-3p, which targets GSTP1 and inhibits proliferation, migration, and invasion.	[140]
	miR-335	Hypermethylation	Leads to decreased miR-335, which targets CRKL and represses proliferation and migration while inducing apoptosis and cell cycle arrest at G0/G1 phase. Another potential target of miR-335 is RASA1, which has reported roles in cell invasion and metastasis.	[141,142]
	miR-495-3p	Hypermethylation	Downregulates miR-495-3p, which regulates ten oncogenic epigenetic modifiers of HDAC2, KDM1A, KDM2B, KDM5B, CREBBP, EP300, MYST3, SMYD3, DNMT1, and MTA1.	[143]
Lung	miR-886-3p	Hypermethylation	Loss of miR-886-3p and consequently reduced levels of PLK1 and TGF-β1, thereby inhibiting cell invasion, migration, and proliferation.	[144]
	miR-34b/c	Hypermethylation	Results in lower levels of miR-34b/c. Functional analysis of miR-34b/c revealed that ectopic expression of the miR-34 family suppressed cell proliferation, invasion, and migration in SCLC cell lines.	[145]
	miR-224	Hypomethylation	miR-224 promoter hypomethylation status was linked to high levels of miR-224, promoting cell proliferation and migration by targeting TNFAIP1 and SMAD4, genes known for their respective proapoptotic and anti-migratory functions in lung cancer.	[146]
	Let-7a-3	Hypomethylation	Re-expression of Let-7a-3 following DAC treatment revealed the involvement of DNA hypomethylation in the regulation of let-7a-3 in lung cancer. Ectopic expression of let-7a-3 was associated with anchorage-independent cell growth.	[147]
	miR-135b	Hypomethylation	Upregulation of miR-135b was observed in highly invasive CLI-5 lung cancer cells. miR-135b promotes cell invasion, tumor growth, and metastasis by targeting LZTS1 and some players of the Hippo signaling pathway.	[148]
	miR-486-5p	Hypermethylation of *ANK1* promoter	No functional studies were performed, but an inverse correlation between the hypermethylated *ANK1* promoter and intronic miR-486-5p expression levels in NSCLC cell lines was reported.	[149]
	miR-126	Hypermethylation of *EGFL7* promoter	Reduces miR-126 levels, which impedes cell invasion in NSCLC by targeting Crk, a key regulator of cell growth, motility, differentiation, and adhesion.	[150,151]
Cervical	miR-124	Hypermethylation	Reduces levels of mature miR-124, which has tumor suppressor activity in cervical cancer.	[152]
	miR-375 and miR-196a-1	Hypermethylation	Downregulation of miR-375 and miR-196a-1 inhibits the proliferation of SiHa cells.	[153]
	miR-181a2/181b2	Hypermethylation	Targets the PIK3R3/Akt/FoxO signaling, and its reduction is associated with poor prognosis and advanced-stage cervical cancer.	[154]
Ovarian	miR-30a/c-5p	Hypermethylation	Inhibits cisplatin resistance and EMT by targeting Snail.	[72]
	miR-133b	Hypermethylation	Targets PKM2, inhibiting the Warburg effect.	[73]
Endometrial	miR-152	Hypermethylation	Targets E2F3, MET, and Rictor.	[155]
	miR-137	Hypermethylation	Targets EZH2 and LSD1 and inhibits tumor growth.	[156]
	miR-129-2	Hypermethylation	Targets the oncogene SOX4.	[157]
Prostate	miR-152-3p	Hypermethylation	Suppresses cell viability and invasion potential.	[158]
	miR-130a	Hypermethylation	Inhibits cell viability, increased apoptosis, and reduced invasive potential of prostate cancer cell lines.	[159]
	miR-34b	Hypermethylation	Decreases cell proliferation, inhibits EMT, and induces apoptosis in PC3 and LNCaP cells.	[74]
	miR-145	Hypermethylation	Sensitizes PC3 cells to irradiation.	[75]
Hepatocellular	miR-148a	Hypermethylation	Reduces cell proliferation and cell cycle progression.	[160]
	miR-142	Hypermethylation	Targets TGF-β, reducing cell viability, proliferation, and angiogenesis.	[161]
	C19MC	Hypomethylation	Observed in high T-stage HCC tumors with high invasive ability.	[162]

## Data Availability

No new data were created or analyzed in this study. Data sharing is not applicable to this article.
